# Thymus Inception: Molecular Network in the Early Stages of Thymus Organogenesis

**DOI:** 10.3390/ijms21165765

**Published:** 2020-08-11

**Authors:** Marta Figueiredo, Rita Zilhão, Hélia Neves

**Affiliations:** 1 Instituto de Histologia e Biologia do Desenvolvimento, Faculdade de Medicina da Universidade de Lisboa, Edifício Egas Moniz, Av. Prof. Egas Moniz, 1649-028 Lisboa, Portugal; mteles1@campus.ul.pt; 2 Departamento de Biologia Vegetal, Faculdade de Ciências, Universidade de Lisboa, 1740-016 Lisboa, Portugal; rmzilhao@fc.ul.pt

**Keywords:** thymus, T/PT common primordium, pharyngeal pouch endoderm, mesoderm, neural crest, molecular network, transcription factors, signaling molecules

## Abstract

The thymus generates central immune tolerance by producing self-restricted and self-tolerant T-cells as a result of interactions between the developing thymocytes and the stromal microenvironment, mainly formed by the thymic epithelial cells. The thymic epithelium derives from the endoderm of the pharyngeal pouches, embryonic structures that rely on environmental cues from the surrounding mesenchyme for its development. Here, we review the most recent advances in our understanding of the molecular mechanisms involved in early thymic organogenesis at stages preceding the expression of the transcription factor Foxn1, the early marker of thymic epithelial cells identity. Foxn1-independent developmental stages, such as the specification of the pharyngeal endoderm, patterning of the pouches, and thymus fate commitment are discussed, with a special focus on epithelial–mesenchymal interactions.

## 1. Introduction

The thymus (T) is an essential component of the adaptive immune system conserved in all vertebrates [1,2]. It is a specialized primary lymphoid organ that supports T-cell (and Natural-Killer cell) development and maturation, and its absence (athymia) results in severe or complete immunodeficiency [3,4,5]. Thymic immunological functions were discovered in 1961 by Jacques Miller when mice thymectomized immediately after birth showed a deficit in a specific type of lymphocytes, that were later called T-lymphocytes [6]. It took, however, two more decades for the immunological properties of central tolerance—the production of self-restricted and self-tolerant T-cells, by eliminating self-reactive T-cells before their export into the periphery—to be attributed to the thymus [7].

## 2. Thymus Composition

**Figure 1:**
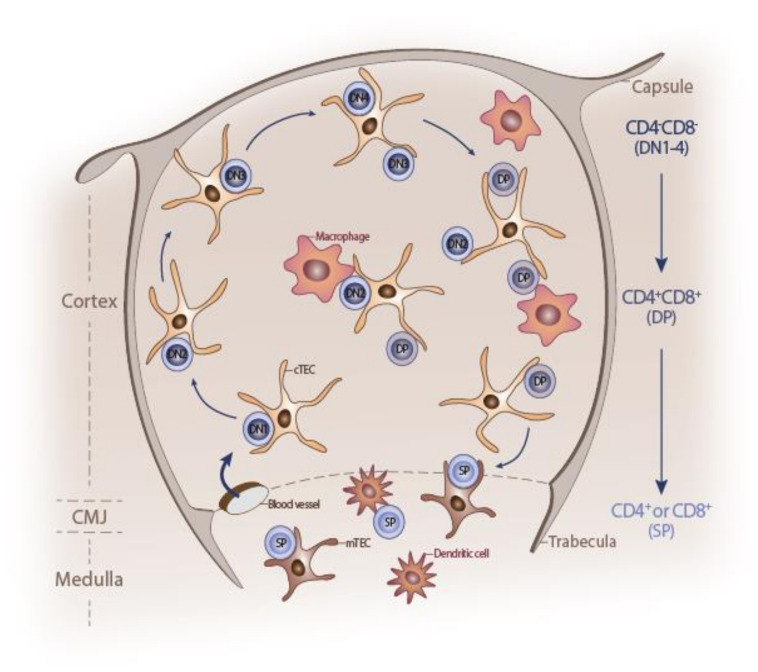
Schematic representation of post-natal thymus. The outer mesenchymal capsule enters the thymus at regular intervals to form trabeculae. Inside, the developing thymocytes are embedded in a three-dimensional network of thymic stroma, mainly composed of thymic epithelial cells (TECs), dendritic cells, endothelial cells, macrophages, and fibroblasts. Each thymus compartment delimited by trabeculae is divided into two histologically distinct regions, the cortex and medulla, separated by the corticomedullary junction (CMJ). Immature hematopoietic progenitors enter the thymus via the vasculature at the CMJ and commit to the T-cell fate. Thymocytes migrate from the CMJ to the subcapsular zone of the cortex, as they differentiate through CD4^–^CD8^–^ double-negative 1-4 (DN1-4) stages to the CD4^+^CD8^+^ double-positive (DP) stage. DP cells interact with cortical TEC (cTEC), differentiate into either CD4^+^ or CD8^+^ single-positive (SP) cells, and migrate back to the CMJ. DP cells positively selected to mature into CD4^+^ or CD8^+^ single positive (SP) cells then cross the CMJ and enter the medulla, where they undergo the final stages of maturation before being exported to the periphery. Self-reactive SP cells are deleted by ‘negative selection’, mediated by thymic dendritic cells and medullary TECs (mTEC).

In young individuals, the thymus contains large numbers of developing lymphoid progenitor cells (LPCs) embedded in a three-dimensional (3D) network of thymic stroma [8]. This multi-component stroma is comprised of a majority of thymic epithelial cells (TECs), dendritic cells, endothelial cells, macrophages, and fibroblasts [9]. The intricate 3D network allows close proximity between the developing LPCs and TECs. TECs exhibit different morphology and gene expression profiles, and provide migratory cues (through the expression of several chemokines) to LPCs’ homing, as well as to the migration of the developing lymphoblasts across the distinct thymic compartments (reviewed in [10,11,12,13,14,15]). TECs subsets also provide distinct microenvironmental niches and signals essential for proper thymocyte differentiation (reviewed in [11,13,16,17]). Cortical TECs are required for commitment, expansion, and positive selection of thymocytes to recognize self-MHC [18], whereas medullary TECs support negative selection, which eliminates potentially autoreactive T-cells, thus, inducing self-tolerance [19] (Figure 1) (reviewed in [20,21,22]).

## 3. Embryonic Origin of the Thymus 

**Figure 2:**
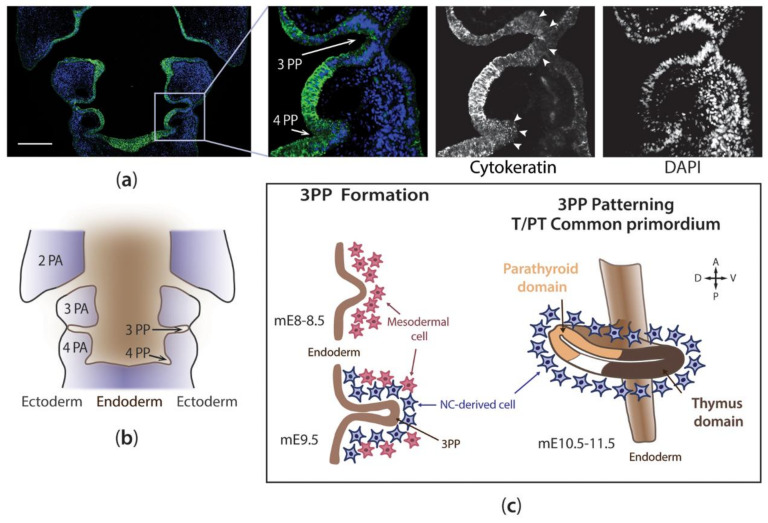
The early stages of thymus development. Hemi-coronal section of the pharyngeal region of a chicken embryo immunodetected with cytokeratin antibody (clone AE1/AE3, which binds to cytokeratin 1–8, 10, 14–16, and 19), at E4, a stage prior to Foxn1 expression. Cytokeratin-positive endoderm and ectoderm cells are observed. The columnar epithelium of the pouches is indicated by white arrowheads in the magnified images (**a**). Schematic representation of coronal section (a), detailing PP and PA locations (**b**). Schematic representation of the cellular interactions between the endoderm and surrounding mesenchymal cells during the early stages of thymus organogenesis in the mouse model (**c**). See main text for details. Color code: Endoderm-, mesoderm-, and NC-derived cells in brown, rose, and blue, respectively. A—anterior; D—dorsal; mE—embryonic day of development in the mouse; NC—neural crest; PA—pharyngeal arch; PP—pharyngeal pouch; P—posterior; T/PT—thymus/parathyroid glands; V—ventral. Scale bar, 200 μm.

The single endodermal germ layer origin of the thymic epithelium (TE) was first demonstrated using the quail-chick chimera system [23]. In chicken, the TE derives from the endoderm of the 3rd and 4th pharyngeal pouches (3/4PP; Figure 2a) [23,24], while in mammals, it derives from the 3PP endoderm (3PP) [25]. The PP are bilateral outpockets of the endoderm, which are in contact with the ectoderm invaginations (the pharyngeal clefts), separating the different pharyngeal arches (PA) (Figure 2b). The PA are composed of a mesodermal core enclosed by NC-derived mesenchyme, an outer ectodermal cover, and an inner endodermal lining (reviewed in [26]).

The thymus shares the same embryological origin with the parathyroid (PT) glands, an organ responsible for the production of the parathyroid hormone (Pth). The endoderm of the 3PP gives rise to the T/PT common primordium in mammals and birds, whereas the 4PP gives rise to the PT primordium only in humans and birds [24,27] (reviewed in [28]). In mouse, the 3PP at E9.5 is a single-cell epithelial layer that is continuous with the pharynx and presents columnar morphology. Between E10.5 and E11.5, the pouches develop to form a multi-layered pseudostratified epithelial structure with a central lumen, which is a residual of the original pouch cavity ([29] and reviewed in [8]). This structural organization is histologically similar to the initial primordium of other epithelial organs undergoing a branching morphogenesis process [29].

By mE11.5, the T/PT common primordium is patterned into thymus (brown) and parathyroid (yellow) domains in the 3PP. The T/PT common primordium develops lined by a thin mesenchymal layer of cardiac neural crest (NC)-derived cells (Figure 2c), which contributes to its development [8,23,30,31,32] and detachment from the pharynx by promoting endodermal apoptotic cell death [33,34,35,36]. An NC-derived capsule is formed around E11.5 [8] and E6.5 (Hamburger and Hamilton-stage29 [37], HH29) [23], in mouse and chicken, respectively. These cells persist in the adult thymus capsule and are a source of pericytes and smooth muscle cells that contribute to the structural support of thymic vasculature [38,39]. Cardiac NC cells are also involved in the separation process (around E12.5 in mouse) of the organ rudiments by cellular intercalation [40] and actively direct the migration of the thymus, while the PT glands appear to be “dragged” during this process [41]. 

During organ separation and migration, the LPCs begin TE colonization [23]. The crosstalk between epithelial cells and developing LPCs is established in a bidirectional manner, which is essential to both T-cell development and TECs maturation [42,43,44,45,46]. TECs differentiation is tightly regulated by the concerted activity of several signaling pathways, transcription factors (TFs), and microRNAs networks ([47], reviewed in [46]).

### The Common Primordium of the Thymus and Parathyroid Glands 

In the PP endoderm, thymic and PT prospective domains can be discriminated by the expression of the organ-specific TFs, forkhead box protein N1 (Foxn1), and glial cells missing homologue 2 (Gcm2), respectively [25]. In the mouse 3PP endoderm, Foxn1 starts to be expressed in the ventral domain at mE11.25 and gives rise to the thymus, while Gcm2 expression starts in the dorsal domain as early as mE9.5 and gives rise to PT glands [25]. In avian species, the 3/4PP endoderm also expresses Gcm2 and Foxn1 in the organ rudiments, but its domains occupy inverted positions along the dorsal–ventral axis when compared to mammals [24,27]. In chicken embryos, the in situ expression of Gcm2 and Foxn1 begins in the 3/4PP endoderm at E3.5 (HH22) and E4.5 (HH25), respectively [24,27]. 

It is possible that the expression of Gcm2 prior to the formation of the T/PT common primordium illustrates an evolutionary legacy, but it may also reflect the need to preserve the PT domain from a thymus fate within the pouch [48]. The expression of Gcm2 and Foxn1 is maintained throughout the development of the organs and after birth, both in mammals and birds [24,25,49]. Increasing evidence has been produced in recent years showing the fine-tuning of Foxn1 levels in the regulation of several processes, such as thymic epithelium differentiation, adult thymus homeostasis, and thymic involution (reviewed in [50]).

- Thymic Epithelial Marker—Foxn1

The Foxn1 gene, originally named winged helix nude (Whn) [51], belongs to the family of winged helix/forkhead TFs. It binds to specific DNA sequences via the evolutionarily conserved forkhead box (Fox) domain, thereby activating its target genes. *Nude* mice with Foxn1 mutation have congenital athymia that results in severe immunodeficiency [49,51]. The name “*nude*” comes from the mutant’s first description in 1966, as these mice exhibited a lack of fur development since birth, distinct from previously described “hairless” mutants [3]. Although Foxn1-deficient mice lack a functional thymus, a thymic primordium is formed and migrates to its final position [49]. However, TECs remain in an early progenitor state and fail to attract T-cell precursors, which remain in the surrounding perithymic mesenchyme [49,51,52,53]. As a consequence, the thymus does not develop its characteristic 3D organization and eventually degenerates into cysts [54]. In agreement, it was recently attributed to Foxn1 the capacity to prevent tubulogenesis of the thymic rudiment [55]. Interestingly, experiments in which unmanipulated pharyngeal endoderm was grafted ectopically showed its ability to give rise to a functional thymus [23,33]. 

This evidence suggests that Foxn1 is required cell-autonomously for TECs differentiation, regulation of branching morphogenesis, and thymus colonization, rather than being responsible for thymus specification [49,51,53,55]. Other factors, such as upstream of Foxn1, must regulate thymus-cell fate decision.

- Parathyroid Epithelial Marker—Gcm2

Gcm2 encodes a TF homologous of the Drosophila gene Gcm [56]. It is the earliest known marker of the PT glands in all higher vertebrates (except for fish, which have no PT glands). Gcm2 deletion in mice results in a lack of PT glands, showing its key importance in PT glands development [57]. Although Gcm2-deficient mice develop a PT-specific domain, they are unable to express Pth and to steadily synthesize other PT-specific markers, such as Ccl21 and CaSR. In these mutants, the primordium undergoes rapid and coordinated apoptosis by mE12.5 [48]. These data highlight Gcm2 as a major regulator of the differentiation and survival of PT precursor cells, but not of PT glands specification [48]. 

## 4. Molecular Regulation of Thymus Early-Organogenesis

**Figure 3:**
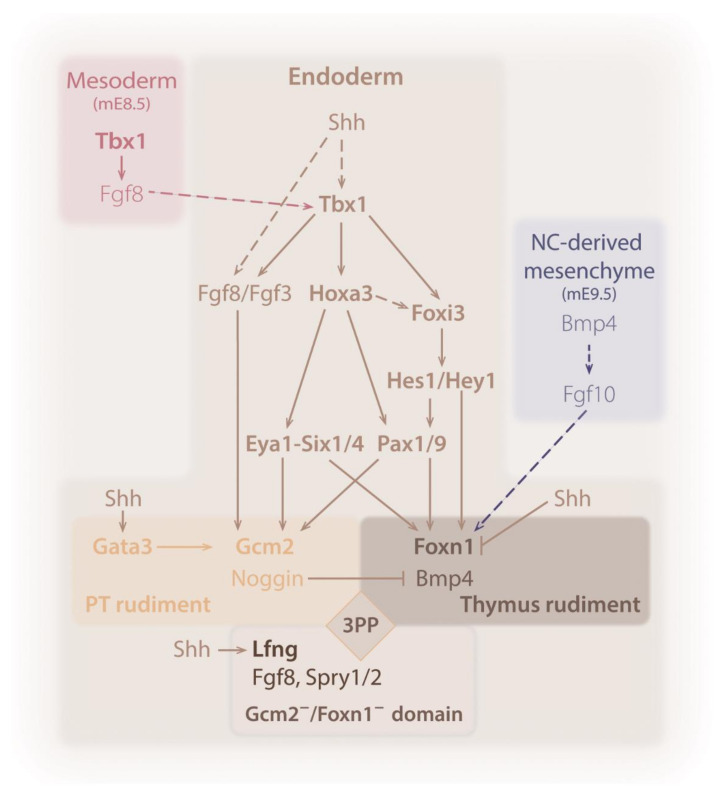
Schematic diagram of potential interactions of factors in the early steps of the formation of the thymus. See main text for details. Color code of the different tissue compartments: Endoderm—from yellow to dark brown; mesoderm—rose; NC mesenchyme—blue. Solid and dashed lines indicate known and hypothetical interactions, respectively. Bold font—transcription factor. Regular font—signaling molecule.

Organogenesis comprises distinct stages regulated by a network of interacting signaling molecules and TFs that ensure correct organ formation. Most of the data on the molecular regulators of/acting on the early stages of T/PT development came from mouse mutants, but several studies in the avian and zebrafish models also added relevant knowledge to this field. The role and expression patterns of the main potential regulators in thymus and PT glands formation are discussed below and summarized in Table 1. A schematic representation of the most relevant signaling networks is detailed in Figure 3.

### 4.1. Factors Implicated in the Morphogenesis of the Pouch

- Retinoic acid

Retinoic acid (RA), the biologically active derivative of vitamin A, was shown to be one of the diffusible mesodermal signals that pattern the posterior pharyngeal endoderm in mice [82], quail [83], and zebrafish [93]. Reduced RA signaling through pharmacologic compounds [82,93], genetic manipulation [84], or retinoid-deficient diet [83] results in the complete absence of the most posterior PP (3-6PP). Moreover, RA was found to positively regulate the expression of early PP-endoderm markers—such as Fgf8, Pax1, and Pax9—and other important factors in pouch formation and development—such as Tbx1 and Hoxa3 [82,83,84,91,94]. Interestingly, blocking RA signaling during pouch formation in zebrafish embryos did not impair pouch specification, but affected the morphogenesis and segmentation of the pouches in a time-dependent manner [93]. The loss of RA signaling was also found to result in NC cells defects, but several studies have shown that RA influence in neural crest outgrowth is secondary to its role in patterning the pharyngeal endoderm [82,84,95]. Taken together, the data suggest that RA is a major regulator in posterior pouch segmentation and formation that subsequently supports NC cells migration.

- T-box 1 and Fibroblast Growth Factor 8

T-box transcription factor 1 (Tbx1) belongs to the evolutionary conserved family of T-box TFs, which share a common DNA-binding domain (designated T-box), and are capable of interacting with other transcriptional factors to regulate the expression of target genes [96]. Tbx1 is one of the genes responsible for the malformations found in DiGeorge syndrome in humans, that includes cardiovascular defects, abnormal facial features, and hypoplasia or aplasia of the thymus and PT glands [90,97,98,99]. During pouch formation (Figure 2c), Tbx1 is expressed in the surface ectoderm overlying the pharynx, the pharyngeal endoderm, and non-NC-derived mesenchyme, later becoming restricted to the PPs endoderm and mesodermal core of the PAs [99,100,101]. With the use of a Tbx1-lacZ reporter gene, it was shown that Tbx1 displays both anterior/posterior and medial/lateral gradients in the developing pharyngeal region [99]. Mice with no Tbx1 display a hypoplastic pharyngeal cavity, with abnormal patterning of the 1PA, hypoplasia of the 2PA, aplasia of the caudal PAs (3-6PAs), and impaired formation of the 2-4PP, that ultimately results in organs aplasia [90,97,99]. The non-segmented caudal pharyngeal apparatus of Tbx1^−/−^ mutant mice suggests that Tbx1 has an important role in pharyngeal region segmentation. However, Tbx1 loss-of-function in zebrafish revealed that the endoderm of the Tbx1 mutant retains, to some extent, segmental characteristics [102]. Tbx1 is required for pouch formation, both in the mouse [103] and zebrafish [102]. Tbx1′s role may be associated with the regulation of cell proliferation, as Tbx1^−/−^ mice mutants have a downregulation of the proliferative activity of endodermal cells [103]. Deletion of Tbx1 exclusively in the pharyngeal endoderm [104,105] or in the non-NC-derived mesoderm of mice [106] recapitulated most of the developmental defects of Tbx1^−/−^ embryos, suggesting that Tbx1 is required in both tissues, and that epithelial–mesenchymal interactions may be relevant in this process (Figure 3). It is interesting to note that Tbx1 has a suppressive role in later stages of organ development, needing to be repressed for the differentiation of TECs [107]. These data suggest a biphasic role of Tbx1 activity in different development windows, which should be supported by the existence of different regulatory mechanisms upstream from Tbx1. 

Increasing evidence has pointed to fibroblast growth factor 8 (Fgf8) as a potential downstream effector of Tbx1 role in PP formation (Figure 3). Fgf8 belongs to the FGFs family, which comprises small proteins generally secreted, which bind to transmembrane tyrosine kinase receptors (FGFRs). These signaling molecules are involved in cell proliferation, differentiation, and survival (reviewed in [108]). Fgf8 is expressed in the pharyngeal endoderm, overlying ectoderm, and non-NC-derived mesenchyme prior to pouch formation, becoming restricted to the PPs endoderm and their respective ectodermal clefts upon their formation [109]. While Fgf8 null mice die at mE8.5 [110], Fgf8 hypomorphic mutants display hypoplasia/aplasia of the 3/4PA and PP (and consequently, of T/PT), suggesting the involvement of Fgf8 in 3/4PP formation [66,67]. These hypomorphic mice display defects in NC cells [66,67] similar to NC cells-ablation phenotype [31], suggesting a role of Fgf8 in the regulation of these cells. In agreement, Fgf8 deletion solely in the pharyngeal endoderm results in normal segmentation of the pharyngeal region [105]. Fgf8 expression is reduced in Tbx1-deleted tissues [104,105,106], and the loss of Fgf8 diminishes the mitotic activity in both the pharyngeal endoderm and mesenchyme [111], suggesting a Fgf8-mediated regulation of proliferation by Tbx1 in the pharyngeal region. In addition, mutant and transgenic rescue experiments in zebrafish embryo revealed that mesoderm-derived Tbx1 is responsible for guiding pouch epithelial outpocketing through Fgf8a, suggesting that Tbx1 may act primarily in the mesenchyme for pouch morphogenesis, and subsequently, in the endoderm for other aspects of pouch cell biology such as their proliferative expansion [102].

### 4.2. Factors Implicated in the 3PP Endoderm Patterning and Early T/PT Development

#### 4.2.1. Transcription Factors

There is growing evidence that a Hox-Eya-Six-Pax regulatory network of TFs is operating during T/PT common primordium specification and differentiation (Figure 3) (reviewed in [112]). These genes, Hoxa3, Eya1, Six1, Six4, Pax1, and Pax9, are expressed at least initially in the 3/4PP endoderm, and the null mutants for each of them have normal pouch formation, but then, fail to form or have hypoplastic organs [64,65,76,78,80,81]. Additionally, Hoxa3 expression is unaltered in each of the single null mutants for the other genes, as well as in Eya1/Six1, Six1/Six4, and Pax1/Pax9 double homozygous embryos [65], placing Hoxa3 upstream of the genetic cascade. It remains to be clarified if Hoxa3 regulates Pax1 and Pax9 independently of Eya1 and Six1. 

More recently, the transcription factor Foxi3 has increased the complexity of the hierarchical cascade of TFs. Foxi3 acts as a downstream effector of Tbx1 and regulates Pax9 [70], while leaving open the question to a possible co-regulation with Hoxa3. Foxi3 is expressed in the 3 PP endoderm and mice homozygous null for Foxi3 fail to form the thymus and parathyroid glands [70]. Other TFs, Nkx2.5, Nkx2.6, Isl1, and Foxg1, are expressed in the 3PP endoderm and identify the thymus-fated cells in a Foxn1-independent manner [74,113]. They remain, however, as potential candidates to the gene regulatory network of Hox-Eya-Six-Pax-Foxi3, as their role in the development of T/PT common primordium and thymic rudiment still requires additional clarification.

- Homeobox Protein A3

Homeobox protein A3 (Hoxa3) belongs to the homeobox family of TFs that are known to play an important role in patterning the anterior–posterior axis of bilaterian embryos [114,115]. In mouse, Hoxa3 is expressed in the 3/4PP endoderm and in the surrounding NC cells from mE8.5 and mE9.5, respectively [77,94]. Hoxa3^−/−^ mice form normal 3/4PP but fail to develop thymus and PT glands, resulting in organs aplasia [36]. The specific Hoxa3 deletion in the endoderm or in NC cells results in small ectopic thymus and PT glands, whereas gene deletion in both tissues mimics the null phenotype, indicating that Hoxa3 expression in either cell type is sufficient for organs formation [36]. Although for many years the Hoxa3^−/−^ phenotype was thought to be due to a failure in the specification of the T/PT common primordium into organ rudiments [76,78,116], it was shown that Foxn1 and Gcm2 expression are initiated, but the primordium undergoes coordinated apoptosis shortly after [36]. Though Hoxa3 is not responsible for 3/4PP identity nor thymus and PT glands-specific gene expression, Hoxa3 protects the organ rudiments from cell death [36].

- Eyes Absent 1 and Sine Oculis Homeobox 1 and 4

Eyes absent homolog 1 (Eya1) is a member of the eyes absent gene family, homolog of the Drosophila eyes absent (Eya) gene [117], and encodes a transcription co-activator that is expressed in the pharyngeal endoderm, NC-derived mesenchyme, and ectoderm from mE9.5 [64]. Eya1 null mutant mice lack thymus and PT glands, and the expression of Foxn1 and Gcm2 was not detected at mE9.5-11.5, suggesting that Eya1 is necessary for early initiation of T/PT organogenesis [64]. In addition, Eya1 was proven to be a canonical activator of sine oculis homeobox 1 (Six1) [118], a member of the Six gene family of TFs homologous to Drosophila sine oculis (so) gene [119]. Eya1 acts synergistically with Six1 to regulate proliferation and survival of organ-specific precursors [118]. In the pharyngeal region, Six1 is co-expressed with Eya1 and its expression is Eya1-dependent [64]. Six1 knockout mice display a phenotype with strong similarities to Hoxa3^−/−^ mice, as the expression of Gcm2 and Foxn1 initiates, but the primordium undergoes apoptosis, leading to the complete disappearance of these glands by mE12.5 [36,65]. The fact that the expression of organ-specific genes initiates in Six1^−/−^ mice, but not in Eya1^−/−^ mutants, also supports the notion that Eya1 acts upstream of Six1 [65]. In addition, the double knockout of Six1 and Six4 (a closely related family member, co-expressed with Six1 in the pharyngeal endoderm) shows a complete absence of Gcm2 and Foxn1 expression, indicating that both proteins act synergistically and downstream of Eya1 to regulate organ primordium-specific gene expression during early T/PT formation [65].

While Eya1^−/−^ mice show no changes in Pax1/9 expression, Eya1^−/−^ Six1^−/−^ embryos were reported to have undetectable Pax1 expression (but unchanged Pax9 expression) in the pouches at mE10.5 [65]. Considering these data, and the fact that Eya1 and Six1 expression is unaltered in Pax1/Pax9 single and double homozygous mutants [65], it is plausible that Eya1 and Six proteins act upstream of Pax1/9. However, it is also possible that they are acting in parallel pathways, both regulated by Hoxa3.

- Paired Box Protein 1, 3, and 9

Pax1 and Pax9 are closely related members of the paired box (Pax) family of TFs [120]. Distinct from the other players of the Hox-Eya-Six-Pax network, their expression is restricted to the pharyngeal endoderm. Pax1 and Pax9 are expressed in the pharyngeal endoderm from mE8.0 in the pharyngeal pouches by mE9.5 and, further in development, in TECs [79,121]. Although these highly homologous genes exhibit overlapping patterns of expression in the pharyngeal region, mRNA levels of Pax9 were observed to be distinctly lower than those of Pax1 [121]. Pax1 null mutant mice have normal initial pouch patterning and organogenesis but display thymic and PT glands hypoplasia along with mild defects in T-cell development [79]. A much more drastic phenotype is observed in Pax9^−/−^ mutants, as the T/PT common primordium fails to detach from the pharynx and develops ectopically as a polyp-like structure within the laryngeal cavity [81]. Although the thymic primordium of the mutant expresses Foxn1 and is colonized by LPCs, it is severely hypoplastic. T-cell development is greatly impaired, and the thymic lobes gradually become filled with apoptotic cells, resulting in highly disorganized rudiments [81]. 

Worth noticing, Pax1/9 expression initiates in Hoxa3^−/−^ mice but fails to be maintained [76], which mimics what is observed for Gcm2 expression in these mice [36]. This points to a potential regulatory network between Hoxa3, Pax1/9, and Gcm2. Although Pax1^−/−^ mice show a reduction in Gcm2 transcript levels, resulting in hypoplastic PT glands, the compound mutants Hoxa3^+/−^ Pax1^−/−^ display an inability in maintaining Gcm2 expression, and the hypoplastic PT rudiment observed at formation eventually disappears [78]. This evidence suggests a Hoxa3-Pax1-Gcm2 regulatory cascade, although the presence of PT glands in Pax1 single mutants indicates the existence of other players under the control of Hoxa3 during PT glands differentiation. 

A potential candidate is Pax9, as both Pax1 and Pax9 binding sites were found to be present in the promoter of the Gcm2 gene [122]. Noteworthy, functional redundancy between Pax1 and Pax9 was reported during vertebral column development in a gene dosage-dependent manner [123]. The fact that Hoxa3^+/−^ Pax1^−/−^ hypoplastic thymus are also ectopic, a feature not observed in Pax1^−/−^ mutants, but characteristic of Pax9^−/−^ mice, also suggests functional redundancy between Pax1/9 in thymus development. Analysis of several Pax1/9 compound mutants has provided further evidence suggesting a gene-dose cooperation between the two genes in the modulation of Foxn1 expression, formation of the rudiment and TECs and T-cell development [124]. 

Pax3, another member of the pair box family, is expressed in NC cells and null mutant mice for this gene (*Splotch* mice) are largely deficient in migratory NC cells [34]. *Splotch* mice display abnormal boundary formation between thymic and PT domains, with enhanced thymic domain and subsequent larger thymus at the expense of the PT glands, which become correspondingly smaller [34].

- Forkhead Boxi3

Recently, Foxi3, which belongs to the Fox TFs family, was shown to act as a downstream effector of Tbx1 in PA segmentation (Figure 3). The loss of Foxi3 in the Tbx1 expressing lineage disrupts segmentation between PA3–4 [70]. Foxi3 is expressed in the epithelia of the PA around the same stages as when Tbx1 is expressed [125]. Foxi3^−/−^ null mutant mouse embryos fail to form endodermal pouches resulting in abnormal PA segmentation and thymus and PT glands aplasia. Additionally, the Tbx1^+/−^ Foxi3^+/−^ double heterozygous mouse embryos had thymus and parathyroid gland defects similar to those observed in deletions of 22q11.2DS in DiGeorge Syndrome patients [70].

- Other Transcription Factors and Cytokines

Several candidate regulators of thymic specification have been proposed based on their expression in the presumptive thymic domain, prior to the expression of Foxn1 (mE11.25) [74]. Foxg1, a member of the forkhead family of TFs, is synthesized in the endodermal region that includes both PT glands and thymic presumptive domains at mE10.5 [74]. One day later, it becomes restricted to the thymic domain [62,74]. The presence of the homeobox proteins Islet1 (Isl1), Nkx2.5, and Nkx2.6 is restricted to the endoderm of the presumptive thymic domain at mE10.5 [74]. Isl1 and Foxg1 continue to be expressed in TECs throughout thymus development, suggesting these factors may have a continuous role in TECs differentiation [74]. Interleukin 7 (IL7), a cytokine required for thymocyte differentiation and survival [126], is also one of the earliest markers of thymus-fated cells [113]. IL7 expression is initiated around mE10.5 and it becomes exclusively expressed in the thymic domain of the pouch by mE11.5 [113]. It should be noted that both Foxg1 and IL7 were shown to be expressed in the thymic rudiment of nude mice [74,113]. To sum up, Nkx2.5, Nkx2.6, Isl1, Foxg1, and IL7 are Foxn1-independent specific early markers of thymus-fated cells [74,113]. However, it remains unclear whether they are involved in the activation of Foxn1 expression, in its maintenance, and/or in other Foxn1-independent aspects of thymus development [74]. It is known that Nkx2.6 null mutant has no apparent phenotypic thymus alterations [127], and the data regarding the other mutants are limited, keeping open the questions as to their role in thymus formation. 

#### 4.2.2. Major Signaling Pathways

One of the main challenges when studying organ development is to reach a comprehensive view of the concerted regulatory actions of the major signaling pathways in development. Besides the number of transcriptional factors described so far, signaling pathways like bone morphogenetic protein (BMP) [24,59,60,61,62,63], fibroblast growth factor (FGF) [66,67,68,69,128,129,130], Wingless-int (Wnt) [92], Notch [73,75,131], and Hedgehog [86,87,88,89], were also shown to be involved in the 3/4PP patterning and the early phases of thymus and PT glands development (Figure 3).

- BMP and FGF Pathways

BMPs are a group of secreted morphogenetic growth factors that belong to the transforming growth factor-β (TGF-β) family. They are involved in embryonic patterning and development, and regulate tissue homeostasis and regeneration [132]. Bmp4 is known to be expressed in the presumptive domain of the thymus before the settlement of high levels of Foxn1 [18], while its antagonist, Noggin, is expressed in the complementary Gcm2-domain [60]. In addition to the endodermal expression, Bmp4 is also expressed in the surrounding mesenchyme [18], including NC-derived cells, and in the ectodermal compartment [60] (Figure 3). 

In the avian model, a sequential expression of Bmp4 and Fgf10 in the mesenchyme was shown to be crucial for the formation of a Foxn1-thymic rudiment. Bmp4 signals are only required through a short period of time, after which Fgf10 expression takes over, sustaining the later development of the endoderm into a Foxn1-thymic rudiment. Concomitant to Fgf10 forthcoming in the mesenchymal compartment, Bmp4 starts to emerge in the pouch endoderm, revealing the fine-tuning of endodermal–mesenchymal interactions that are essential for T/PT early development [24]. Mice genetically modified to have Noggin expression under the Foxn1 promoter showed the requirement of endodermal Bmp4 signaling for the maintenance of Foxn1 expression. In addition, the upregulation of Foxn1 transcripts in fetal thymic organ cultures upon Bmp4 treatment stressed the role of Bmp4 in regulating Foxn1 expression [133].

Besides the described roles for Fg8, the analysis of mice with a hypomorphic or null allele of Fgf8 [67] and mice with specific deletion of Fgf8 in both the endoderm and ectoderm [68] have confirmed Fgf8 involvement in the development of the thymus and PT glands. FGF-related molecules, including Fgf8 and Fgf10, are expressed in the posterior region of the 3PP and in the surrounding mesenchyme, previous to Foxn1 expression [24,69]. The deletion of two members of the Sprouty (Spry) class of FGF antagonists—Spry1 and Spry2—led to a delay in Foxn1 expression and a reduction in the Gcm2-domain, which later results in both organs hypoplasia [69]. Interestingly, Bmp4 expression is also downregulated in the thymic domain, supporting the notion that FGF signaling in the posterior domain of the pouch may regulate the initiation of the patterning events of the 3PP in mouse [69]. These alterations, caused by enhanced expression of FGF targets, are partially suppressed by genetic reduction in Fgf8 [69].

- Wnt pathway

The Wnt family of secreted glycolipoproteins controls several cellular processes during development and in adult homeostasis, such as cell proliferation, polarity, and fate specification. Wnt4 expression was shown to precede the appearance of Foxn1 in the thymic primordium, and overexpression of Wnt4 in TEC lines induces Foxn1 transcription, highlighting its potential role in Foxn1 regulation [92]. 

Further evidence on the regulation of Foxn1 expression by Wnt and BMP pathways came from the hair follicle development in mice. Treatment of cultured mouse skin with Wnt5a induced Foxn1 expression [134], while the overexpression of Noggin in the skin reduced Foxn1 mRNA levels in hair follicles [135]. It has to be noted that Wnt5b, a paralog of Wnt5a, is expressed in the 3PP of mouse embryos prior to strong Foxn1 expression [65,92]. However, Wnt5b’s precise domain of expression in the pouch and its potential link with Foxn1 remain unknown.

- Notch Signaling

Notch signaling is involved in multiple cellular events like fate decision, proliferation, survival, and differentiation, during development and in the post-natal life. Notch effects are highly dependent on dose, timing, and context (reviewed in [136,137,138,139,140]). The most well-known involvement of Notch signaling related to thymus is, undeniably, in T-cell lineage commitment and maturation. Numerous studies have shown that Notch is required for the late stages of thymus development, not only in the cross-talk between TECs and LPCs (reviewed in [46]), but also throughout T-cell development—in T-cell commitment [141], in the choice between αβ and γδ TCR and between CD4 and CD8 lineages (reviewed in [142,143,144]). Despite all the knowledge gathered over the years concerning the fine-tuning of Notch signaling in thymic functions, there is still very limited evidence of its actions in early stages of thymus formation.

Several of the Notch signaling receptors, ligands, modulators, and target genes are expressed in the pharyngeal arch region at stages of T/PT common primordium development [70,75,131,145]. In chicken embryos, Notch1 and the ligand Delta1 are faintly expressed in endoderm and neighboring cells of the 3PP. At the same time, another Notch ligand and a modulator, Jagged1 (Jag1) and Lunatic Fringe (Lfrg), are strongly expressed in complementary domains of the 3PP endoderm. Lfrg is detected in the posterior/median territory of the pouch, a region excluded from the T/PT common primordium. Considering that Lfng is known to inhibit Jag1-mediated signaling and to potentiate Notch1 activation via the Delta1 ligand [146], it is conceivable there is a preferential activation of Notch via Lfng/Delta1 in the posterior/median domain of the pouches, which, in turn, may act as a regulatory center. The Notch targets, Hes1 and Hey1, are downstream of Tbx1 and Foxi3 in the development of the pharyngeal structures [70,145], and Hes1 is required for organs migration to their final destination [131]. Other Notch target genes, Hes5.1, Hes6.1, and Gata3, are expressed in the pharyngeal region of avian embryos [75]. In particular, Gata3 is expressed in T/PT common primordium and afterward is restricted to the Gcm2-domain when Foxn1-domain is established, both in chicken and mouse [73,74,75]. Gata3^−/−^ mice embryos lack Gcm2 expression and do not develop the T/PT primordia, while Gata3^+/−^ heterozygotes display smaller T/PT primordia with fewer Gcm2-expressing cells [73]. Gata3 was shown to bind directly to the Gcm2 promoter region and to upregulate its expression in the mouse [73]. In avian embryos, the pharmacological inhibition of Notch activity in the pharyngeal arch region at the common primordium stage reduces Gcm2-domain compromising PT development [75] (Figure 3). Additionally, it reduces Pax1 expression and transiently abolishes Foxn1 expression, suggesting that Notch signaling may be upstream in the cascade of Pax1-Foxn1 [75]. 

Besides the described roles for Tbx1 and Foxi3, the analysis of their null mutant mice revealed their regulatory action of Notch signaling, as they present downregulation of the expression of the Notch ligand, Jag1, and the Notch targets, Hes1 and Hey1, in the 3 PP-cleft region [70] (Figure 3). The compound heterozygous mutants for Tbx1/Foxi3, further show reduction in Gcm2, Foxn1, and Pax9 expression in the 3PP endoderm [70]. Considering the recent data, we may envisage new players in a distinct Tbx1-Foxi3-Notch-Pax-Foxn1/Gcm2 regulatory network, operating during T/PT common primordium specification and differentiation.

- Hedgehog Signaling

The Hedgehog (Hh) pathway is a major paracrine regulator of many fundamental processes in development including cell proliferation, survival, and differentiation, cell fate, stem cell maintenance, and tissue polarity. Hh ligands act as morphogens, signaling both at short range and over many cell diameters [147]. The synthesis of Hh ligand and receptor, sonic hedgehog (Shh), and of the protein patched homolog 1 (Ptch1), are mainly restricted to the anterior pouches region, both in chicken and mouse embryos [75,87,88]. By the time Gcm2 starts being expressed, Shh is present throughout the pharyngeal endoderm, with the exception of the pharyngeal pouches [75,87,88]. Ptch1 transcripts co-localize with those of Shh and are also present in mesenchymal cells surrounding Shh-expressing endoderm.

Hh signaling is involved in cranio-facial and neck morphogenesis [26] and the Shh null mice display loss of Noggin/Gcm2 domain, while Bmp4/Foxn1 domain is expanded in the 3PP [87]. This abnormal patterning of the common primordium results in the lack of PT glands [87] and in thymic functional defects [86] (Figure 3). Interesting to note, the genetic deletion of Smoothened (Smo) in the endoderm or in the adjacent NC cells of mouse embryos did not prevent Gcm2 expression, suggesting that each tissue alone is sufficient to promote PT glands development [89].

Similar changes were observed in chicken embryos treated with a pharmacological inhibitor of Hh signaling [88]. These changes are accompanied by a reduction in Gata3 expression in the Gcm2-domain and expansion of Foxn1-domain into the Lunatic fringe (Lfng)-expressing domain [75]. The domain of the Notch-modulator Lfng, is excluded from the common primordium in the 3PP of chicken embryos. The concomitant reduction in Lfng and Fgf8 transcripts in the same territory suggests that this domain may be involved in the regulation of T/PT common primordium development, in an Hh-dependent manner [75]. A putative Shh-Fgf8-Lfng network may be envisaged involving distinct signaling centers located in the endoderm of the pharynx and within the pouches. In other biological contexts, Lfng is known to respond to Fgf8 signals [148] and Fgf8 has been shown to respond to Shh produced by the pharyngeal endoderm during arch patterning [149] (Figure 3). 

A decade ago, a Shh-Tbx1-Gcm2 regulatory network was proposed [48] based on the fact that Shh signaling regulates Tbx1 expression in the pharyngeal region of mouse and chicken embryos [100,150] together with the observation that Tbx1 regulates Gcm2 expression in PT glands domain [48,91,99,151]. Moreover, Tbx1′s suppressive role for thymus fate specification was later confirmed, though Tbx1 ectopic expression is not sufficient to induce Gcm2 expression in the thymic domain [107]. Similarly, constitutive activation of Hh signaling in the endoderm of mice results in an expanded Tbx1-domain with partially suppressed Foxn1 expression with no expansion of the Gcm2-domain [89]. The data, thus, confirmed Tbx1 as a target of Shh signaling in the patterning of the 3PP, and Shh and Tbx1 as negative regulators of thymus development. It also suggested that other players, independent of Shh, must be involved in PT glands fate specification.

- Eph/ephrin Signaling

The Eph (erythropoietin-producing hepatocellular carcinoma) receptors and their ligands, ephrins, have a pleiotropic role in several developmental processes and act as important mediators adult tissue homeostasis (reviewed in [152]). In particular, Eph and ephrins are involved in numerous processes of thymus development and functions (reviewed in [153]).

Mice with the ephrin-B2 ligand specifically excluded from NC-derived cells exhibit an ectopic thymus, with an apparently normal initial development evincing typical Hoxa3 expression in the 3rd pharyngeal region and migration of NC cells [41]. When ephrin-B2 is conditionally deleted on the thymic medullary compartment at later stages, thymocyte–TEC interactions are affected and tridimensional organization and differentiation of medullary TECs display abnormal features with epithelial cysts formation [154]. Future endeavors may unravel the role of Eph/ephrin signaling in the early stages of thymus development.

## 5. Conclusions

We describe the recent advances made in our understanding of molecular signals and cellular interactions responsible for regulating early embryonic events crucial for the emergence of the thymus rudiment. The genetic, biological, and molecular approaches using vertebrate model organisms such as the mouse, chick, and zebrafish have greatly contributed to clarifying pouch patterning, the formation of the T/PT common primordium, and thymus rudiment. However, many questions remain, and further research is needed to improve knowledge in the field. We believe that elucidating molecular and cellular interactions underlying early stages of thymus organogenesis will pave the way for future strategies to restore thymic function in humans and to produce thymic organoids for use in regenerative therapies.

## Figures and Tables

**Table 1:** Key signaling molecules and TFs implicated in T/PT early development.

Gene	Relevant Expression Pattern	Relevant Role	Reference (s)
Bmp4	T presumptive domain; NC-derived mesenchyme, surface ectoderm.	PP patterning; early T/PT development; organs separation and migration; regulation of Foxn1 expression.	[24,58,59,60,61,62,63]
Eya1	PP endoderm; NC-derived mesenchyme; Surface ectoderm.	PP patterning and outgrowth.	[64,65]
Fgf8	PP and pharyngeal endoderm; non-NC-derived mesoderm; Surface ectoderm.	PP formation and patterning, possible role in guiding pouch epithelial outpocketing.	[66,67,68,69]
Foxi3	PP endoderm; surface ectoderm	PA segmentation; T/PT development	[70]
Foxn1	T rudiment.	TEC differentiation.	[25,49,71,72]
Gata3	PP endoderm; organ rudiments	Possible role in PP patterning and survival; PT differentiation and survival	[73,74,75]
Gcm2	PT rudiment.	PT differentiation.	[48,57]
Hoxa3	PP endoderm; NC-derived mesenchyme	PP specification, T/PT primordium formation and survival	[76,77,78]
Noggin	PT rudiment; Mesenchyme	PP patterning, opposing Bmp signaling	[24,60]
Pax1	PP endoderm	Early T/PT development, possible regulation of Foxn1 expression	[78,79]
Pax3	NC-derive mesenchyme	Organs boundary formation	[34]
Pax9	PP endoderm	PP development, T/PT primordium formation and separation; possible regulation of Foxn1 expression.	[80,81]
RA	Mesenchyme surrounding the pharyngeal endoderm	Posterior PP segmentation and formation	[82,83,84,85]
Six1/4	Surface ectoderm, PP endoderm, NC-derived mesenchyme	Early T/PT formation and survival	[65]
Shh	Pharyngeal endoderm, but excluded from PP endoderm	PP patterning and early PT development	[75,86,87,88,89]
Tbx1	Pharyngeal endoderm and presumptive PT domain; non-NC-derived mesenchyme; surface ectoderm.	Pharyngeal region segmentation; PP formation; possible involvement in promoting PT fate/suppressing T fate	[48,90,91]
Wnt4	PP endoderm; mesenchyme	Possible regulation of Foxn1 expression	[92]

NC—neural crest; PP—pharyngeal pouch; PT—Parathyroid glands; T—thymus; TEC—thymic epithelial cell.

## Abbreviations

3D	three-dimensional
CK	cytokeratin
HH	Hamburger and Hamilton
LPCs	lymphoid progenitor cells
mE	embryonic day of development in the mouse
MHC	Major Complex of Histocompatibility
NC	neural crest
Pth	parathyroid hormone
PA	pharyngeal arch
PP	pharyngeal pouch
PT	parathyroid
T	thymus
TE	thymic epithelium
TECs	thymic epithelial cells
TFs	transcription factors
